# Establishment of a reverse transcription–recombinase polymerase amplification–lateral flow dipstick method for the dual detection of Israeli acute paralysis virus and chronic bee paralysis virus

**DOI:** 10.3389/fmicb.2024.1389313

**Published:** 2024-05-15

**Authors:** Li Sun, Yu Cheng, Dongliang Fei, Yueyu Ma, Mingxiao Ma, Ming Li

**Affiliations:** ^1^College of Animal Husbandry and Veterinary Medicine, Jinzhou Medical University, Jinzhou, China; ^2^Experimental Animal Center of Jinzhou Medical University, Jinzhou, China

**Keywords:** IAPV, CBPV, RPA, LFD, dual detection

## Abstract

**Introduction:**

As an important social insect, honey bees play crucial roles in agricultural production, sustainable development of agricultural production, and the balance of the natural environment. However, in recent years, Israeli acute paralysis virus (IAPV) and chronic bee paralysis virus (CBPV), the main pathogens of bee paralysis, have continuously harmed bee colonies and caused certain losses to the beekeeping industry. Some beekeeping farms are located in wild or remote mountainous areas, and samples from these farms cannot be sent to the laboratory for testing in a timely manner, thereby limiting the accurate and rapid diagnosis of the disease.

**Methods and results:**

In this study, we used a reverse transcription–recombinase polymerase amplification–lateral flow dipstick (RT–RPA–LFD) method for the dual detection of IAPV and CBPV. RPA primers and LFD detection probes were designed separately for their conserved genes. Primers and probes were screened, and the forward and reverse primer ratios, reaction times, and temperatures were optimized. According to the results of the optimization tests, the optimal reaction temperature for RT–RPA is 37°C, and when combined with LFD, detection with the naked eye requires <20 min. The developed RPA–LFD method specifically targets IAPV and CBPV and has no cross-reactivity with other common bee viruses. In addition, the minimum detection limit of the RT–RPA–LFD method is 101 copies/μL.

**Conclusion:**

Based this study, this method is suitable for the detection of clinical samples and can be used for field detection of IAPV and CBPV.

## 1 Introduction

Honey bees, the most widespread and economically significant pollinator in the world, play a crucial role in agricultural production. Approximately one-third of global crop production relies on bees for pollination. Honey bees not only directly affect the yield and quality of various crops but also produce bee products that contain several bioactive ingredients with biological characteristics such as anti-inflammatory, antibacterial, anticancer, antioxidative, antiapoptotic, antidiabetic, antiallergic, hypolipidemic, hypotensive, and immune regulatory effects (Ullah et al., 2021; Bakour et al., 2022; El-Seedi et al., 2022; Giampieri et al., 2022; Sanyal et al., 2023); moreover, they provide substances for use as raw materials and medicines (Bakour et al., 2022; El-Seedi et al., 2022; Giampieri et al., 2022). Bee products have become increasingly popular among consumers. In addition, bees protect and restore the biodiversity of local wild plants and are widely used in polluted areas to obtain valuable information about the environmental presence of pollutants and their impact on human and ecosystem health. However, in recent years, bee paralysis caused by Israeli acute paralysis virus (IAPV) and chronic bee paralysis virus (CBPV) has become one of the important factors leading to colony collapse syndrome and substantial losses of worker bees (Dittes et al., 2020).

IAPV and CBPV are both single- and positive-stranded RNA viruses that have become prevalent worldwide, particularly in Europe, America, and Asia (Mullapudi et al., 2016; Ghorani et al., 2017; Cirkovic et al., 2018; Budge et al., 2020; Fernandez de Landa et al., 2020; Tlak Gajger et al., 2021). Moreover, clinical cases of IAPV and CBPV infection have continued to increase in recent years (Dittes et al., 2021), which has seriously threatened the development of the apiculture industry. Currently, the detection methods for IAPV and CBPV include symptom diagnosis, electron microscopy observation, serological testing, and routine molecular biology testing such as reverse transcription–polymerase chain reaction (RT–PCR) and RT–quantitative PCR (RT–qPCR). However, based on these methods, the diagnosis of symptoms is not sufficiently precise, electron microscopy observation and serological testing are time-consuming and laborious, and conventional molecular biology diagnosis faces problems such as the need for special experimental equipment and operational difficulties and thus can only be used for laboratory testing. However, because bees are raised according to their honey source, some beekeeping farms are located in wild or remote mountainous areas, where it is challenging to send infected bee samples to the laboratory for testing and diagnosis in a timely manner, often leading to the spread of the disease throughout the entire bee colony and causing substantial losses. Therefore, there is an urgent need for developing a method that can rapidly detect these two viruses in the nature.

As a new type of isothermal amplification technology, recombinase polymerase amplification (RPA) allows the reaction to proceed at a constant temperature under the action of three enzymes without the need for an expensive thermocycler. Reactions can proceed under conditions of 23°C–45°C, with an optimal temperature range of 37°C–42°C (i.e., amplification can occur at room temperature and detection can be achieved within 20 min) (Olaf et al., 2006). Lateral flow test strips (LFD) (Shahin et al., 2018) represent an extremely simple and rapid detection method that usually requires only 5–10 min to obtain results; moreover, the results are visible to the naked eye. RPA–LFD not only avoids the possibility of cross-contamination but also eliminates the need for bulky laboratory equipment and cumbersome handling steps; thus, it is suitable for rapid on-site detection. In this study, we described a detection method involving RPA and LFD. We designed probes and primers for IAPV and CBPV, respectively, and optimized the reaction system for their rapid detection. The sensitivity and specificity of our detection method were also evaluated. We successfully established an RT–RPA–LFD detection method that can simultaneously detect IAPV and CBPV and evaluated its application in the detection of infected worker bee samples. Beekeepers can use this method to detect infected bee colonies and achieve early and rapid diagnosis of IAPV and CBPV.

## 2 Materials and methods

### 2.1 Viruses and primary reagents

The purified samples of IAPV, CBPV, acute bee paralysis virus (ABPV), Chinese sacbrood virus (CSBV), deformed wing virus (DWV), and black queen cell virus (BQCV) were stored in our laboratory. *Escherichia coli* DH5α and FlyCut^®^ HindIII were purchased from TransGen Biotech (Beijing, China). The RNA Constant Temperature Rapid Amplification Kit (Colloidal Gold Test Strip Type) was purchased from Amplification Future (Jiangsu, China). HybriDetect 2-T Dipsticks were purchased from Milenia Biotec GmbH (Giessen, Germany). The worker bees were provided by the Experimental Animal Center of Jinzhou Medical University.

### 2.2 Standard plasmid construction

Based on CBPV (GenBank: KU950353.1, RNA1) and IAPV (GenBank: MZ269472.1) isolated in our laboratory, genes with highly conserved regions (CBPV RNA-dependent RNA polymerase (RdRp) and IAPV RdRp) of approximately 300 bp were selected as target genes, and HindIII restriction sites were inserted upstream and downstream of the targets. IAPV and CBPV were synthesized and inserted into the pUC57 plasmid by Sangon Biotech Shanghai Co., Ltd. (Shanghai, China) to obtain a quality control plasmid.

### 2.3 Design and optimization of primers and probes

According to the principle of RPA primer design, primers were designed and screened using the primer-BLAST function of the National Center for Biotechnology Information. We designed four pairs of primers and one probe for the conserved region of the IAPV RdRp gene (6195–6,495 nt, GenBank: MZ269472.1). We selected the conserved region of the CBPV RNA1 RdRp gene (1607–1957 nt, GenBank: KU950353.1) and designed five pairs of primers and one probe for this conserved region (Supplementary Table 1). RPA was performed using the primers listed in Supplementary Table 1. Buffer A (29.5 μL) was mixed with 1.2 μL each of forward (F) and reverse (R) primers (final concentration of 10 μM), 1 μL of the control plasmid, 0.6 μL of the probe, 47.5 μL of diethylpyrocarbonate (DEPC) water, and 2.5 μL of buffer B. After thorough mixing, the samples were isothermally amplified at 37°C for 20 min. The amplified products were analyzed via 1.5% agarose gel electrophoresis. The optimal primers and probes were then labeled and prepared. The probe was prepared as follows: 5-carboxyfluorescein (FAM) at the 5′-end, tetrahydrofuran (THF) in the middle, and a C3-spacer at the 3′-end of the probe. The reverse primer was prepared as follows: biotin at the 5′-end of CBPV-R and digoxin at the 5′-end of IAPV-R. All primers and probes were synthesized by Sangon Biotech Co., Ltd. (Shanghai, China).

### 2.4 Selection of the optimal primer ratio for RT–RPA–LFD

To eliminate false-positive results in RPA–LFD, we adjusted the proportion of upstream and downstream primers in the reaction system. When the total number of primers remained unchanged, the forward and reverse primers were allowed to react in a ratio of 1:1 to 6:1 to determine the optimal primer ratio. HybriDetect 2 T Dipsticks (Milenia Biotec GmbH, Giessen, Germany) were inserted into the PCR tube, and the results were interpreted upon the appearance of the control line.

### 2.5 Optimization of the RT–RPA–LFD detection temperature

The established reaction system comprised 29.5 μL of buffer A, 2.4 μL of primer-IAPV, 2 μL of RNA, 0.6 μL of probe-IAPV, 2.4 μL of primer-CBPV, 0.6 μL of probe-CBPV, 47.5 μL of DEPC water, and 2.5 μL of buffer B. RT–RPA–LFD reactions were performed at 10°C, 15°C, 20°C, 25°C, 30°C, 37°C, 42°C, and 50°C. The experimental results were analyzed to determine the detection temperature range.

### 2.6 Optimization of the RT–RPA–LFD reaction time

Using 37°C as the optimal reaction temperature, the reaction was terminated at 1, 3, 5, 10, 15, 20, 25, and 30 min to determine the optimal and minimum detection time.

### 2.7 RT–RPA–LFD specificity experiment

Using previously stored purified of IAPV, CBPV, ABPV, CSBV, DWV, and BQCV as templates, RT–RPA–LFD reaction was performed under optimal conditions.

### 2.8 Sensitivity determination of RT–RPA–LFD reaction

Following the method of Wang et al. (2016), recombinant plasmids pET28a-MS2-IAPV harboring the IAPV RdRp gene fragment and recombinant plasmids pET28a-MS2-CBPV harboring CBPV RdRp gene fragment were synthesized using armored RNA technology. pET28a-MS2-IAPV and pET28a-MS2-CBPV plasmids were expressed and purified to prepare IAPV-VLPs and CBPV-VLPs. The IAPV-VLP was diluted by 10-fold in nuclease-free water to obtain a series of concentrations ranging from 1 × 10^7^ to 1 × 10^0^ copies/μL. The CBPV-VLP was diluted by 10-fold in nuclease-free water to obtain a series of concentrations ranging from 1 × 10^7^ to 1 × 10^0^ copies/μL. These solutions were used as the reaction templates for RPA–LFD and stored at −20°C before use. The RPA–LFD reaction was performed under the previously described optimal conditions.

### 2.9 Clinical sample testing

Sixty worker bee samples were tested using RT–RPA–LFD, and the results were verified via RT–qPCR (Morfin et al., 2023). The coincidence rate between the results of RT–RPA–LFD and RT–qPCR was obtained, and the accuracy of the former method was determined.

## 3 Results

### 3.1 Selection of optimal primers for RT–RPA–LFD

**Figure 1:**
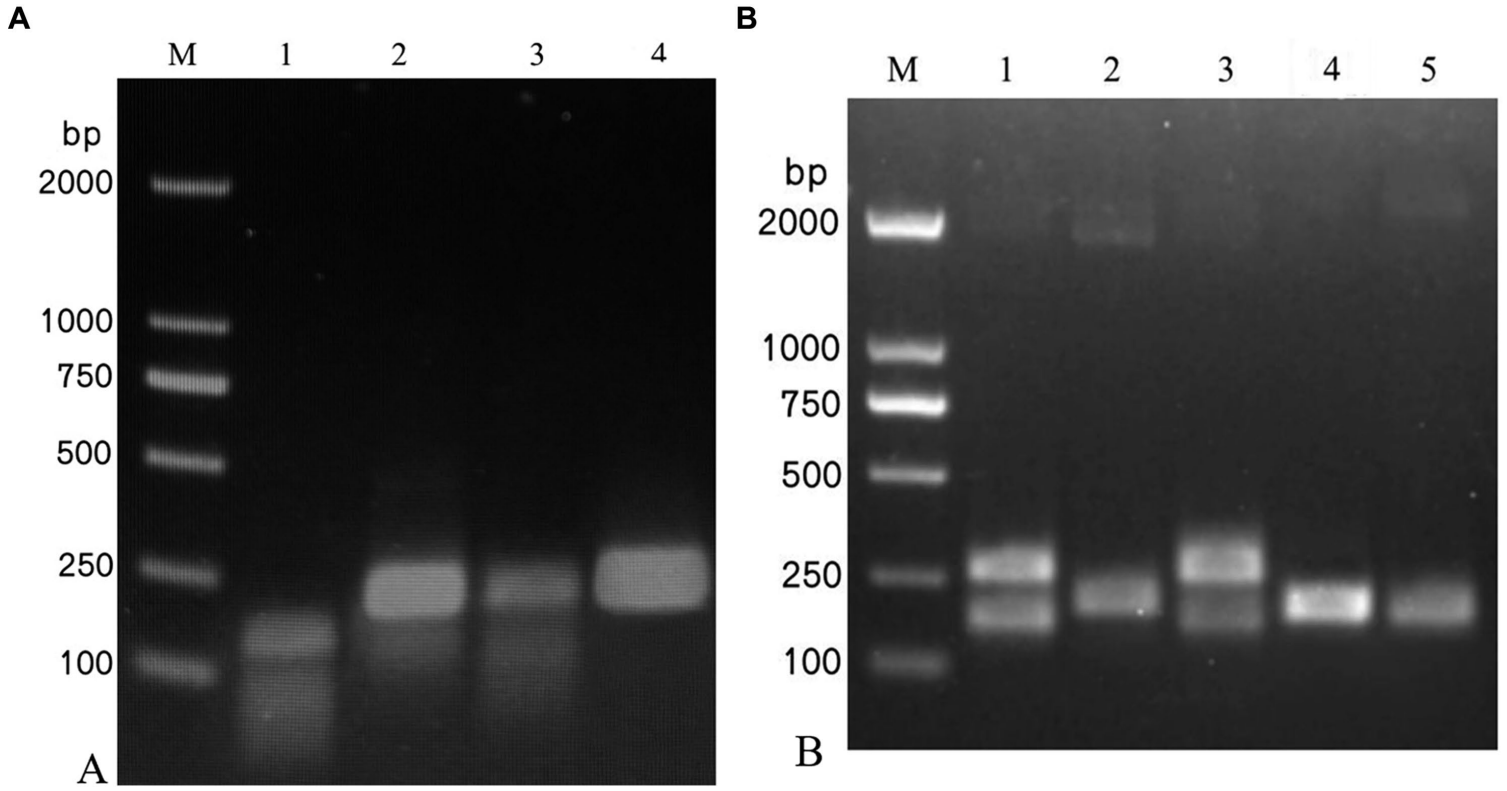
Screening of the optimal primers for detection. A 1.5% agarose gel electrophoresis of RPA products was performed, which revealed different primer combinations. **(A)** IAPV. **(B)** CBPV. M: 2,000 bp DNA marker; 1: primer 1, 2: primer 2, 3: primer 3, 4: primer 4, 5: primer 5.

Among the four pairs of IAPV-specific primers and five pairs of CBPV-specific primers designed, IAPV-F4/IAPV-R and CBPV-F4/CBPV-R4 showed the brightest and clearest target bands under identical conditions without nonspecific bands (Figure 1), indicating their higher amplification efficiency than that of other primer pairs. Therefore, IAPV-F4/IAPV-R4 and CBPV-F4/CBPV-R4 were selected as the optimal primers for the RT–RPA–LFD reaction.

### 3.2 Optimal primer ratio for RT–RPA–LFD

**Figure 2:**
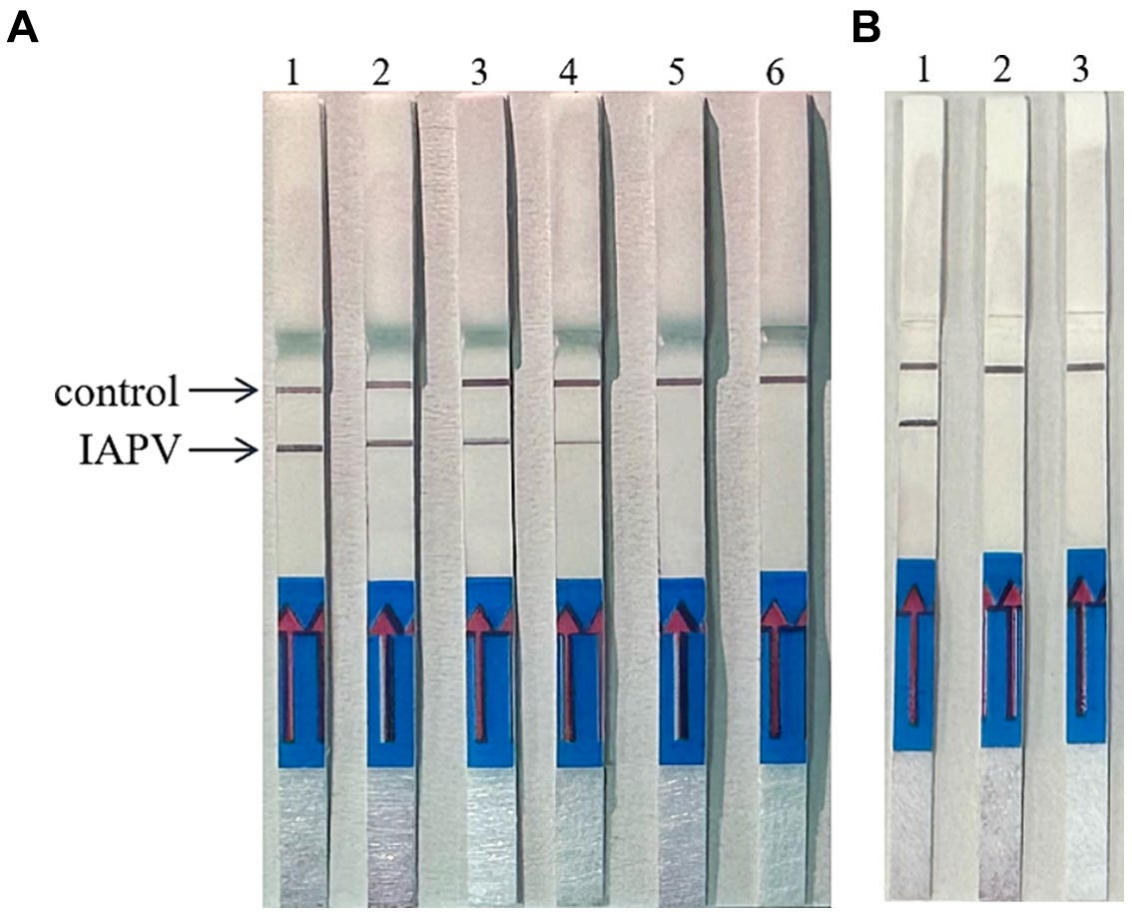
The optimal primer ratio for IAPV. The forward and reverse primers were allowed to react in a ratio of 1:1 to 6:1 to determine the optimal primer ratio. **(A)** 1–6, 1:1–6:1. **(B)** Reaction results using the optimal primer ratio of 5:1, 1: IAPV, 2: Healthy worker bees, 3: water.

**Figure 3:**
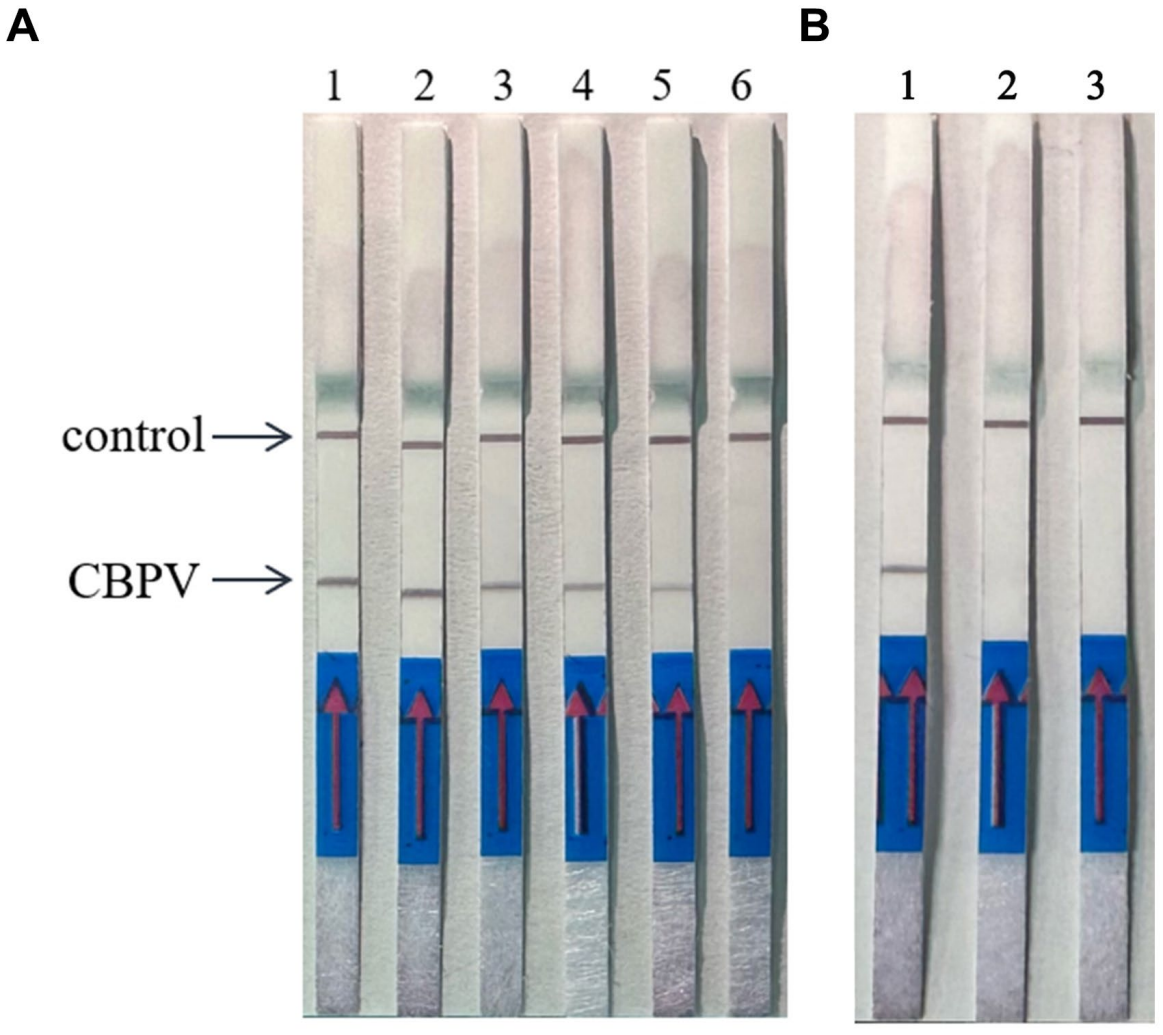
The optimal primer ratio for CBPV. The forward and reverse primers were allowed to react in a ratio of 1:1 to 6:1 to determine the optimal primer ratio. **(A)** 1–6, 1:1–6:1. **(B)** Reaction results using the optimal primer ratio of 6:1, 1: CBPV, 2: Healthy worker bees, 3: water.

When the ratio of upstream and downstream primers in IAPV and CBPV was 5:1 (Figure 2) and 6:1 (Figure 3), respectively, no false-positive bands were detected. Therefore, the abovementioned ratios were selected for upstream and downstream primers in IAPV and CBPV.

### 3.3 Optimization of the RT–RPA–LFD detection temperature

**Figure 4:**
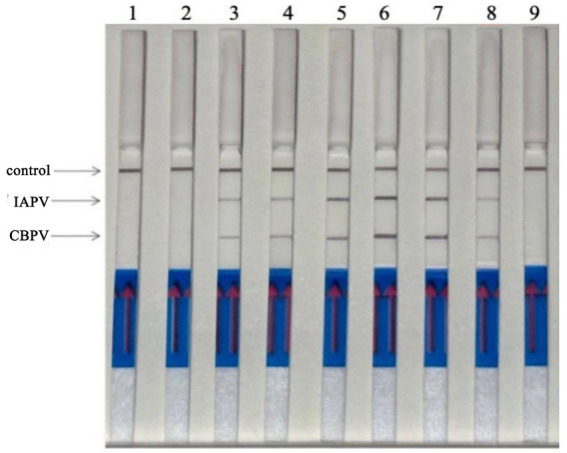
Optimization of RT–RPA–LFD detection temperature. The reactions were performed at 10°C–50°C, the color of the test line was most intense at 37°C. 1: 10°C, 2: 15°C, 3: 20°C, 4: 25°C, 5: 30°C, 6: 37°C, 7: 42°C, 8: 50°C.

By expanding the temperature range, the upper and lower limits of the allowed reaction detection temperature as well as optimal temperature were tested, as shown in Figure 4. When the reaction temperature was 20°C, the test line was colored. As the temperature increased, the color of the test line gradually deepened, with the most intense color appearing at 37°C. When the temperature reached 42°C, the color intensity of the test line decreased, and the color was extremely light at 50°C. This phenomenon may have occurred due to the inactivation of enzymes in the reaction system caused by high temperature. The optimal reaction temperature was determined to be 37°C.

### 3.4 Optimal reaction time for RT–RPA–LFD

**Figure 5:**
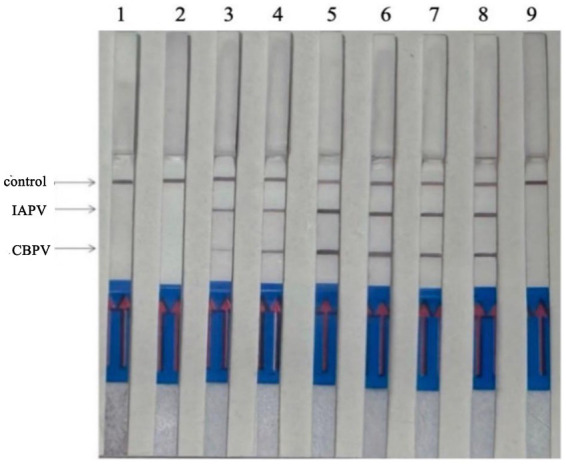
Optimization of RT–RPA–LFD reaction time. 1: 1 min, 2: 3 min, 3: 5 min, 4: 10 min, 5: 15 min, 6: 20 min, 7: 25 min, 8: 30 min.

Reaction time is a key factor in RT–RPA–LFD detection. The reaction time was optimized using the optimal primer ratios and a reaction temperature of 37°C (Figure 5). LFD detection occurred after 5 min of reaction initiation, and the LFD test line began to show color. As time progressed, the color intensity of the LFD test line gradually increased. The color was most intense at 15 min, and no further color intensification was observed after this time point. Thus, we determined that 15 min was the optimal reaction time.

### 3.5 Specificity of the RT–RPA–LFD reaction

**Figure 6:**
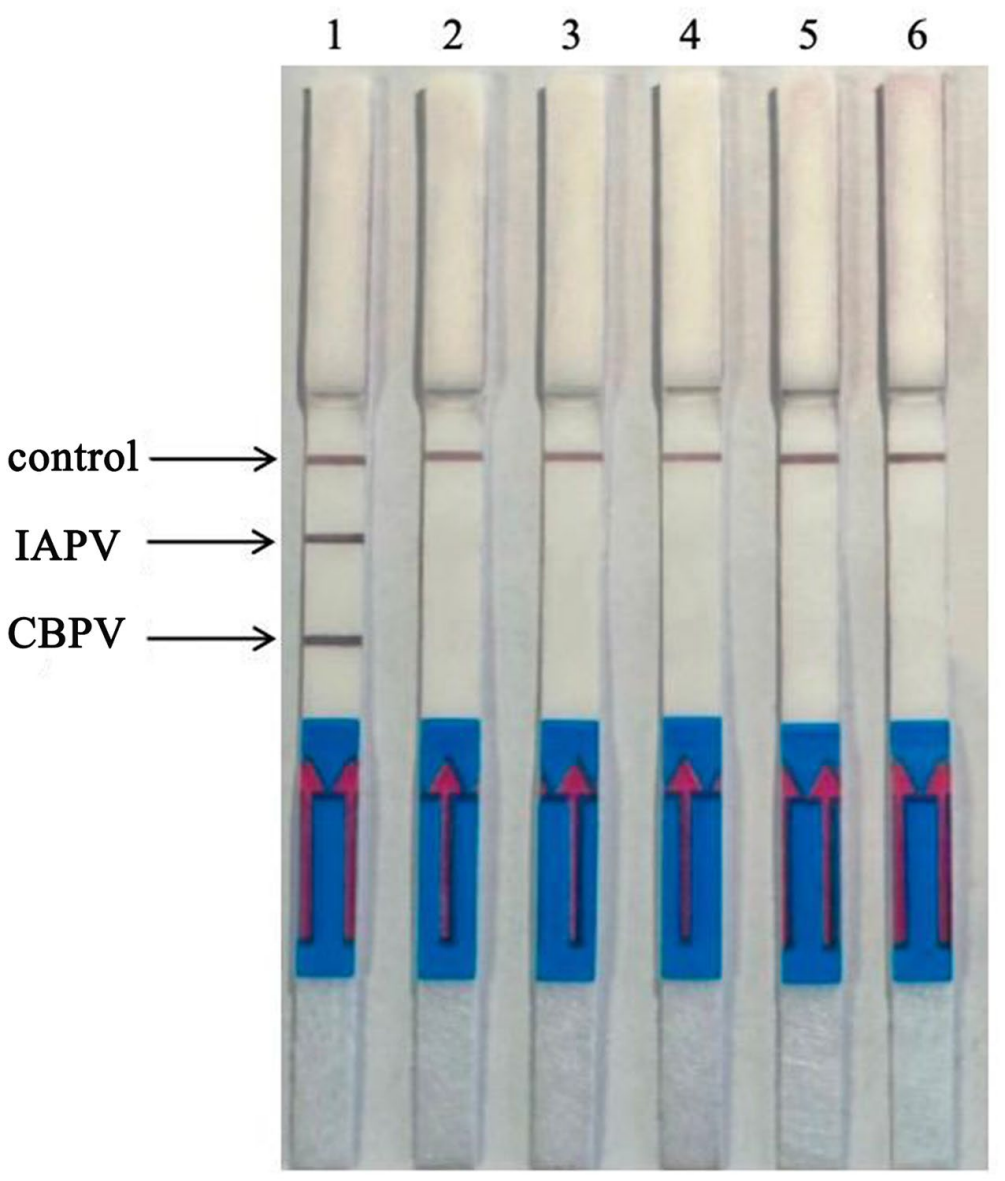
Specificity of the RT–RPA–LFD reaction. Six bee viruses were used to test the specificity of the RPA–LFD reaction, only IAPV and CBPV showed colors on the testing line. 1: IAPV and CBPV, 2: ABPV, 3: CSBV, 4: DWV, 5: BQCV, 6: negative control.

Six bee viruses were used to test the specificity of the RT–RPA–LFD reaction under optimal experimental conditions (Figure 6). The LFD results showed that for all viruses, except for IAPV and CBPV, the LFD produced only control lines, indicating negative results. In contrast, the test lines for IAPV and CBPV showed a distinct color. Therefore, the results revealed that the developed RT–RPA–LFD test was specific for IAPV and CBPV.

### 3.6 Sensitivity determination of the RT–RPA–LFD reaction

**Figure 7:**
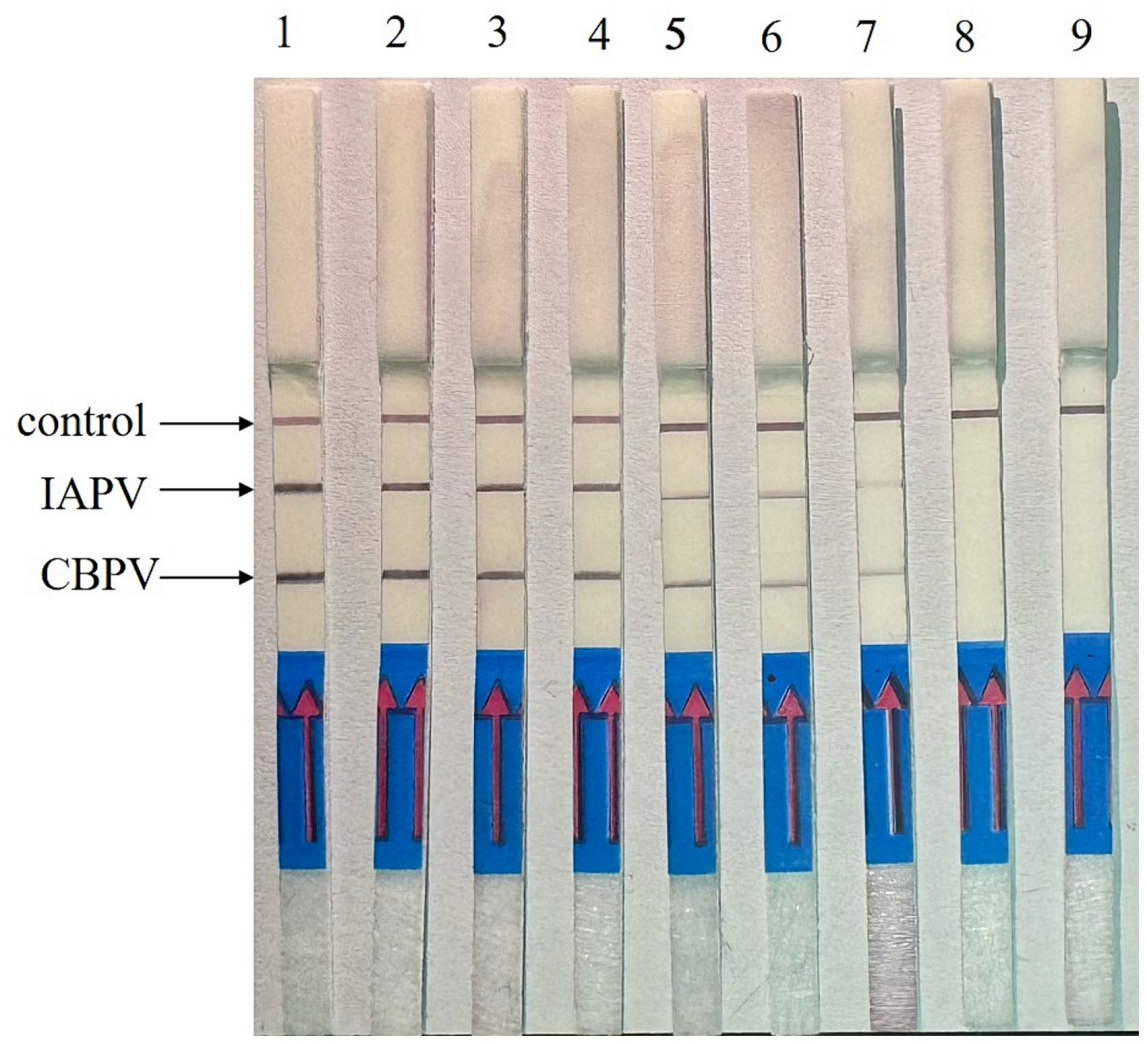
Sensitivity determination of the RT–RPA–LFD reaction. To determine the detection limit of the RT–RPA–LFD assay, 10-fold serial dilutions of IAPV-VLPs and CBPV-VLPs were performed from 1 × 10^7^ to 1 × 10^0^ copies/μL. IAPV: 1–8: 1 × 10^7^–1× 10^0^ copies/μL, 9: negative control. CBPV: 1–8: 1 × 10^7^–1 × 10^0^ copies/μL, 9: negative control.

Experiments were performed using IAPV-VLPs and CBPV-VLPs to evaluate the detection sensitivity of the RT–RPA–LFD reaction. The lowest detection limit for both IAPV and CBPV was 1 × 10^1^ copies/μL (Figure 7).

### 3.7 Clinical sample testing

A total of 60 suspected clinical samples were tested, and the RT–RPA–LFD test results were consistent with those of the RT–qPCR test with 100% accuracy: 26 samples were positive and 34 were negative for IAPV; 13 samples were positive and 47 were negative for CBPV. The results revealed a mixed infection of two samples (Table 1, Supplementary Figure S1). The results indicated the high accuracy of the RT–RPA–LFD detection method, encouraging its use for clinical testing.

**Table 1:** Analysis of detection results for clinical samples using RT–RPA–LFD and qPCR.

Method	Number of samples	IAPV		CBPV		IAPV+ CBPV
		Positive	Negative	Positive	Negative	Positive
RT-RPA-LFD	60	26	34	13	47	2
RT-qPCR	60	26	34	13	47	2

## 4 Discussion

Compared with PCR, RPA technology has the advantages of constant reaction temperature, minimal requirement of equipment (metal or water bath), shorter reaction time (<30 min), and rapid detection suitable for simple outdoor conditions. In this study, the RPA–LFD method was developed for rapid visual detection of IAPV and CBPV. To ensure the normal progression and specificity of the reaction, a probe and multiple pairs of primer combinations were designed based on the conserved regions of IAPV and CBPV genes, respectively, and the most suitable primers and probes for the amplification reaction were screened. To achieve simultaneous detection of IAPV and CBPV, the detection probe was labeled with FAM, and downstream primers were labeled with digoxin and biotin. To eliminate false-positive results and ensure the accuracy of the results, we analyzed the proportion of upstream and downstream primers and ultimately revealed that false-positive results could be eliminated when the ratio of upstream to downstream primers was 5:1 for IAPV and 6:1 for CBPV. The RT–RPA–LFD assay established in the present study had a wide detection temperature range, ranging from 20°C to 50°C, and the optimal reaction temperature was 37°C. The RT–RPA–LFD demonstrated remarkable sensitivity and specificity (10^4^ copies/μL). In addition, the RT–RPA–LFD method showed no cross-reactivity with other common bee viruses. The clinical sample test results were consistent with those of the RT–qPCR test, with high accuracy. Therefore, the newly developed RT–RPA–LFD method can be used for clinical testing.

Currently, the main detection methods for IAPV and CBPV include RT–PCR (Blanchard et al., 2012; Cagirgan and Yazici, 2020) and RT–qPCR (Morfin et al., 2023), which require complex equipment and can only be conducted in a laboratory setting. The newly established RT–RPA–LFD assay does not require complex experimental equipment but only a metal bath and a portable LFD, overcoming the limitations of traditional detection methods, such as prolonged reaction times, complex steps, and extensive requirements of instruments and equipment.

The IAPV and CBPV dual RT–RPA–LFD detection method established in this study can be conducted within a short time and has high sensitivity. Combined with a rapid RNA extraction kit, field detection of IAPV and CBPV can be completed within 30 min. The test results can be directly interpreted through test strips, providing accurate and reliable means for the rapid diagnosis of IAPV and CBPV infection in the field. This facilitates early diagnosis, providing an early warning for the prevention and control of IAPV and CBPV, reducing the losses caused by infection of bee colonies and providing technical support for the healthy development of the bee industry.

## Data availability statement

The datasets presented in this study can be found in online repositories. The names of the repository/repositories and accession number(s) can be found in the article/Supplementary material.

## Author contributions

LS: Methodology, Writing – original draft, Writing – review & editing, Data curation, Project administration, Validation. YC: Data curation, Methodology, Writing – review & editing, Validation. DF: Methodology, Validation, Writing – review & editing. YM: Data curation, Methodology, Validation, Writing – review & editing. MM: Methodology, Writing – review & editing. ML: Methodology, Writing – review & editing, Writing – original draft, Data curation.

## References

[ref1] Bakour M. Laaroussi H. Ousaaid D. el Ghouizi A. Es-Safi I. Mechchate H.. (2022). Bee bread as a promising source of bioactive molecules and functional properties: an up-to-date review. Antibiotics 11:203. doi: 10.3390/antibiotics11020203, PMID: 35203806 PMC8868279

[ref2] Blanchard P. Regnault J. Schurr F. Dubois E. Ribière M. (2012). Intra-laboratory validation of chronic bee paralysis virus quantitation using an accredited standardised real-time quantitative RT-PCR method. J. Virol. Methods 180, 26–31. doi: 10.1016/j.jviromet.2011.12.005, PMID: 22207079

[ref3] Budge G. E. Simcock N. K. Holder P. J. Shirley M. D. F. Brown M. A. van Weymers P. S. M.. (2020). Chronic bee paralysis as a serious emerging threat to honey bees. Nat. Commun. 11:2164. doi: 10.1038/s41467-020-15919-0, PMID: 32358506 PMC7195492

[ref4] Cagirgan A. A. Yazici Z. (2020). Development of a multiplex RT-PCR assay for the routine detection of seven RNA viruses in *Apis mellifera*. J. Virol. Methods 281:113858. doi: 10.1016/j.jviromet.2020.113858, PMID: 32205181

[ref5] Cirkovic D. Stevanovic J. Glavinic U. Aleksic N. Djuric S. Aleksic J.. (2018). Honey bee viruses in Serbian colonies of different strength. PeerJ. 6:e5887. doi: 10.7717/peerj.5887, PMID: 30479890 PMC6240340

[ref6] Dittes J. Schäfer M. O. Aupperle-Lellbach H. Mülling C. K. W. Emmerich I. U. (2020). Overt infection with chronic bee paralysis virus (CBPV) in two honey bee colonies. Vet Sci. 7:142. doi: 10.3390/vetsci7030142, PMID: 32972032 PMC7559786

[ref7] Dittes J. Schierling A. Aupperle-Lellbach H. Grassinger J. M. Mülling C. K. W. Emmerich I. U. (2021). Chronische-Bienenparalyse-virus – quo vadis? Auftreten in Bayern und Betrachtung von Therapiemaßnahmen [chronic bee paralysis virus - quo vadis? Incidence in Bavaria and consideration of therapeutic measures]. Tierarztl. Prax. Ausg. G Grosstiere Nutztiere 49, 326–335. doi: 10.1055/a-1580-8257 34666368

[ref8] El-Seedi H. R. Eid N. Abd el-Wahed A. A. Rateb M. E. Afifi H. S. Algethami A. F.. (2022). Honey bee products: preclinical and clinical studies of their anti-inflammatory and immunomodulatory properties. Front. Nutr. 8:761267. doi: 10.3389/fnut.2021.761267, PMID: 35047540 PMC8762236

[ref9] Fernandez de Landa G. Revainera P. Brasesco C. di Gerónimo V. Plischuk S. Meroi F.. (2020). Chronic bee paralysis virus (CBPV) in south American non-Apis bees. Arch. Virol. 165, 2053–2056. doi: 10.1007/s00705-020-04697-1, PMID: 32556548

[ref10] Ghorani M. Ghalyanchi Langeroudi A. Madadgar O. Rezapanah M. Nabian S. Khaltabadi Farahani R.. (2017). Molecular identification and phylogenetic analysis of chronic bee paralysis virus in Iran. Vet. Res. Forum. 8, 287–292, PMID: 29326786 PMC5756247

[ref11] Giampieri F. Quiles J. L. Cianciosi D. Forbes-Hernández T. Y. Orantes-Bermejo F. J. Alvarez-Suarez J. M.. (2022). Bee products: an emblematic example of underutilized sources of bioactive compounds. J. Agric. Food Chem. 70, 6833–6848. doi: 10.1021/acs.jafc.1c05822, PMID: 34974697 PMC9204823

[ref12] Morfin N. Macías-Macías J. O. Guzman-Novoa E. (2023). Viral quantification in bee samples using synthetic DNA sequences with real-time PCR (qPCR). Methods Mol. Biol. 2610, 57–66. doi: 10.1007/978-1-0716-2895-9_5, PMID: 36534281

[ref13] Mullapudi E. Přidal A. Pálková L. de Miranda J. R. Plevka P. (2016). Virion structure of Israeli acute bee paralysis virus. J. Virol. 90, 8150–8159. doi: 10.1128/JVI.00854-16. PMID: 27384649, PMID: 27384649 PMC5008081

[ref14] Olaf P. Colin H. W. Derek L. S. Armes N. A. (2006). DNA detection using recombination proteins. PLoS Biol. 4:e204. doi: 10.1371/journal.pbio.0040204 PMID: 16756388, PMID: 16756388 PMC1475771

[ref15] Sanyal A. Ghosh A. Roy C. Mazumder I. Marrazzo P. (2023). Revolutionizing the use of honeybee products in healthcare: a focused review on using bee pollen as a potential adjunct material for biomaterial functionalization. J. Funct. Biomater. 14:352. doi: 10.3390/jfb14070352, PMID: 37504847 PMC10381877

[ref16] Shahin K. Ramirez-Paredes J. G. Graham H. Jimena B. L. Adams A. Weidmann M. (2018). Development of arecombinase polymerase amplification assay for rapid detection of *Francisella noatunensis* subsporientalis. PLoS One 13:e0192979. doi: 10.1371/journal.pone.0192979, PMID: 29444148 PMC5812721

[ref17] Tlak Gajger I. Šimenc L. Toplak I. (2021). The first detection and genetic characterization of four different honeybee viruses in wild bumblebees from Croatia. Pathogens 10:808. doi: 10.3390/pathogens10070808, PMID: 34202101 PMC8308666

[ref18] Ullah A. Tlak Gajger I. Majoros A. Dar S. A. Khan S. Kalimullah H. S. A.. (2021). Viral impacts on honey bee populations: a review. Saudi J Biol Sci. 28, 523–530. doi: 10.1016/j.sjbs.2020.10.037, PMID: 33424335 PMC7783639

[ref19] Wang S. Liu Y. Li D. Zhou T. Gao S. Zha E.. (2016). Preparation and evaluation of MS2 bacteriophage-like particles packaging hepatitis E virus RNA. FEMS Microbiol. Lett. 363, 363, doi: 10.1093/femsle/fnw221 27664054

