# Supplementary material for: Establishment of a reverse transcription–recombinase polymerase amplification–lateral flow dipstick method for the dual detection of Israeli acute paralysis virus and chronic bee paralysis virus
Source: Front Microbiol. 2024 May 15;15:1389313. doi: 10.3389/fmicb.2024.1389313 (PMC11137664; doi:10.3389/fmicb.2024.1389313)
Supplement: Supplementary file 1 [file Table_1.DOCX]

| Name Primers and probes Sequence (5’ to 3’) | | |
| --- | --- | --- |
| CBPV | CBPV-F1 | 5’- AAACCTAGTAACATCGATCGAAACCAGAGTGAT -3’ |
|  | CBPV-R1 | 5’- TTTGGGCTTCACAATAATCAGATTCGGTGTCT -3’ |
|  | CBPV-F2 | 5’- TTCCAGCAATGACAATGCAAAACCTAGTAACAT -3’ |
|  | CBPV-R2 | 5’- CATCCCATTCTTTGGCAAAATTTCTCCAGTCC -3’ |
|  | CBPV-F3 | 5’- AAAACCTAGTAACATCGATCGAAACCAGAGTGA -3’ |
|  | CBPV-R3 | 5’- TTTGGGCTTCACAATAATCAGATTCGGTGTC -3’ |
|  | CBPV-F4 | 5’- ACAATGCAAAACCTAGTAACATCGATCGAAACC -3’ |
|  | CBPV-R4 | 5’- CCATCCCATTCTTTGGCAAAATTTCTCCAGTCC -3’ |
|  | CBPV-F5 | 5’- CAATGCAAAACCTAGTAACATCGATCGAAACC -3’ |
|  | CBPV-R5 | 5’- ATCCCATTCTTTGGCAAAATTTCTCCAGTCC -3’ |
|  | Probe-CBPV | 5’-GGACAGTATCTAGTGGCGATGCCCAACCTG/THF/CTCAACACAGGCAACA -3’ |
| IAPV | IAPV-F1 | 5’- CCCACTTTGTATGGACACAATTCTTGARA -3’ |
|  | IAPV-R1 | 5’- CATTTGCATATGCTCGGTCAATTATTTTAG -3’ |
|  | IAPV-F2 | 5’- CCACTTTGTATGGACACAATTCTTGARA -3’ |
|  | IAPV-R2 | 5’- TCACATATAGTATTCCAGAAATCGCTCCTG -3’ |
|  | IAPV-F3 | 5’- TTGTATGGACACAATTCTTGARATGCCAAA -3’ |
|  | IAPV-R3 | 5’- CACATATAGTATTCCAGAAATCGCTCCTG -3’ |
|  | IAPV-F4 | 5’- CTTTGTATGGACACAATTCTTGARATGCC -3’ |
|  | IAPV-R4 | 5’- CATATAGTATTCCAGAAATCGCTCCTGA -3’ |
|  | Probe-IAPV | 5’-ATTGTGAAAATGCAATTATGGAGCTTTCCATG/THF/ATGAAGAGAGCGTTT -3’ |

**Supplementary Table 1** Primers and probes used in this study
