# Supplementary figures and images for: Establishment of a reverse transcription–recombinase polymerase amplification–lateral flow dipstick method for the dual detection of Israeli acute paralysis virus and chronic bee paralysis virus
Source: Front Microbiol. 2024 May 15;15:1389313. doi: 10.3389/fmicb.2024.1389313 (PMC11137664; doi:10.3389/fmicb.2024.1389313)

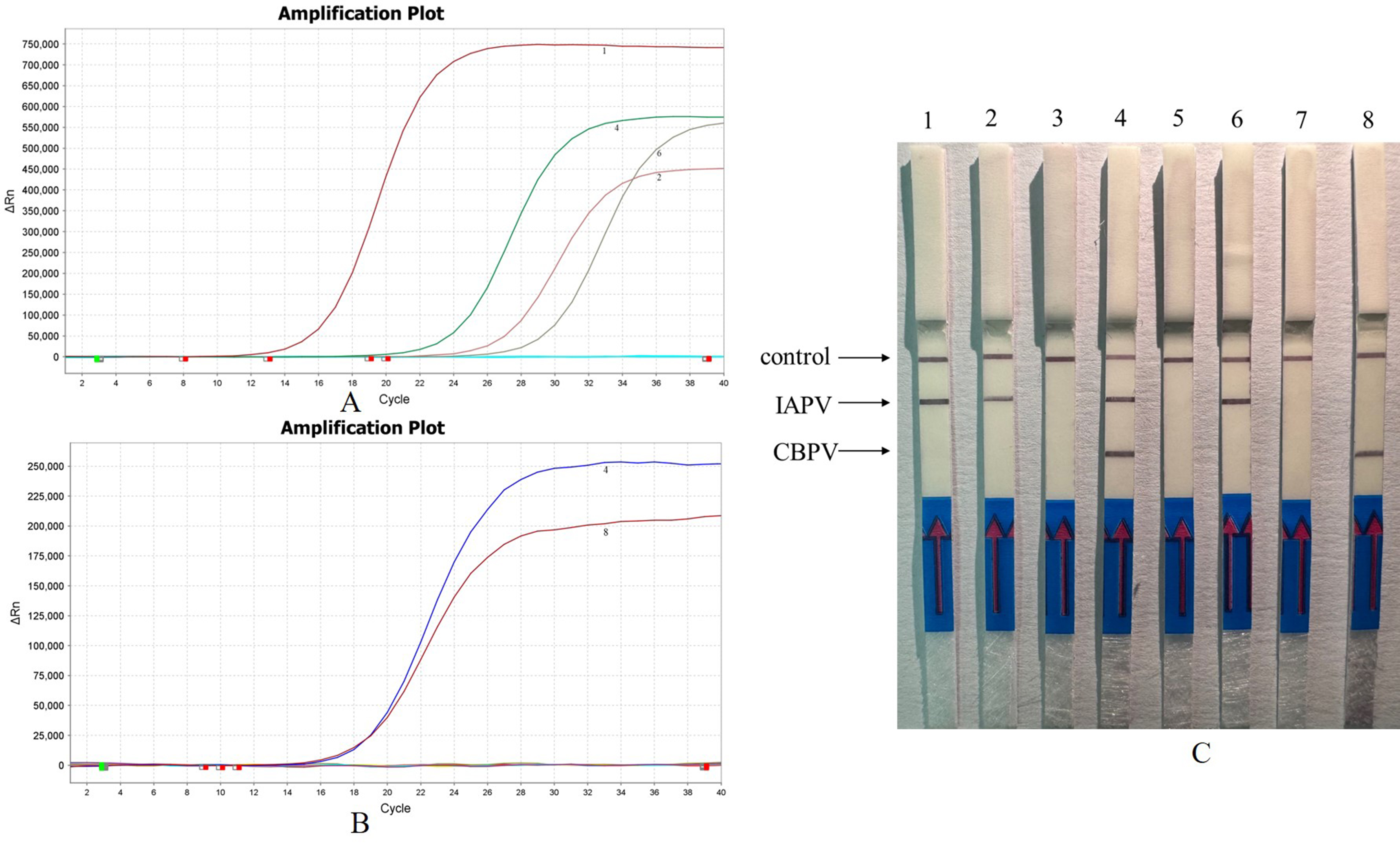

Supplement: Supplementary file 2 [file Image_1.JPEG]
